# Cooling-rate induced softening in a colloidal glass

**DOI:** 10.1038/s41598-017-17271-8

**Published:** 2017-12-04

**Authors:** Yunzhuo Lu, Zhihua Zhang, Xing Lu, Zuoxiang Qin, Jun Shen, Yongjiang Huang, Peter K. Liaw

**Affiliations:** 1 School of Materials Science and Engineering, Dalian Jiaotong University, Dalian, 116028 People’s Republic of China; 2 School of Materials Science and Engineering, Tongji University, Shanghai, 201804 People’s Republic of China; 3 School of Materials Science and Engineering, Harbin Institute of Technology, Harbin, 150001 People’s Republic of China; 4 Department of Materials Science and Engineering, The University of Tennessee, Knoxville, TN 37996 USA

## Abstract

Contrary to crystalline solids, amorphous solids always become softer when vitrifying the melts under higher cooling rates. Understanding this phenomenon is of utmost importance in providing a basis for the mechanical-performance control of amorphous solids. However, the underlying mechanisms leading to this cooling-rate-induced softening of amorphous solids have remained elusive, especially the dynamic reasons are neglected. Here, we use a colloidal glass as the model system to directly study this issue. Shear modulus is used as the representative parameter to monitor the stress-bearing properties of colloidal glass. The space-spanning immobile particles, whose population is sensitive to the cooling rate, are found to make the dominant contribution to the shear modulus. The rapid solidification induced softening of colloidal glass is observed to originate from fewer immobile particles formed at higher cooling rates.

## Introduction

One of the effective ways of hardening crystalline solids is to rapidly solidify the melts into a solid state^1^. For instance, the surface hardness and wear resistance of crystalline metals can be significantly improved by remelting the surface with electron or laser beams and then rapidly cooling^2,3^. However, for amorphous solids, the effect of cooling rate on the stress-bearing properties is reversed. Many results have proven that the rapid solidification can bring about the softening of amorphous solids^4–6^. For example, the hardness and elastic modulus of the Zr_50_Cu_50_ bulk metallic glass were found to be reduced by rapid solidification^4^. This cooling rate induced softening in amorphous solids is always rationalized with the help of configurationally-looser atomic packing or higher defect concentration^7,8^. However, a complete understanding of the underlying mechanisms is still challenging, especially the dynamic reasons are neglected.

A metastable amorphous solid is not homogeneous and isotropic, but rather is heterogeneous concerning its dynamics^9–11^. This dynamical heterogeneity refers to the appearance of spatially correlated domains of mobile and immobile particles^12,13^. The dynamical heterogeneity is central to the evolution of many properties of amorphous solids. It is also a central aspect in our present understanding of the cooling-rate-induced softening in amorphous solids. It can be easily imagined that the correlated clusters of mobile particles cannot bear a stress, and, therefore, contribute to the softening of amorphous solids. Instead, the hardening of amorphous solids must result from transiently immobile particles, which may percolate across the sample and can support a stress. Therefore, the softening of amorphous solids induced by rapid solidification may derive from the smaller regions of transiently-immobilized particles. The only indirect evidence for the correlation between immobile particles and the softening of amorphous solids comes from the computer simulations that find the shear modulus of the Cu_64_Zr_36_ glass reduces with decreasing the amount of “solid-like” clusters with higher energy barrier of transforming^14^. Unfortunately, there has been no direct experimental proof of this hypothetical scenario. Moreover, it is difficult to directly observe this scenario within atomic and molecular solids due to the small length and time scales that characterize the localized atomic motion.

By contrast, colloidal glass of micrometer-sized hard spherical particles can serve as an ideal model to study this possible scenario, since the larger size and concomitant slower time scale of colloidal particles make them much more experimentally accessible^15,16^. These colloidal particles can be directly observed in real time and their three-dimensional (3D) positions can be determined accurately by high-speed confocal microscopy. Subsequent image analysis enables us to track the trajectories of individual particle, providing an accurate picture of interrelationship between dynamical heterogeneity and glass softening. For instance, Conrad *et al*. imaged the individual particles near the colloidal glass transition using confocal microscopy, and successfully connected the spatially correlated clusters of the system to their stress-bearing properties^11^.

In the present work, we build a colloidal glass with a cooling-rate gradient along its height as the model to directly study the cooling rate induced softening of amorphous solids. Shear modulus, *μ*, is used as the representative parameter to monitor the stress-bearing properties of the colloidal glass. We find that the space-spanning immobile particles, whose amount is controlled by the cooling rate, indeed give rise to the shear modulus. The rapid-solidification-induced softening of colloidal glass can be attributed to fewer immobile particles formed at higher cooling rates. The origins for the large shear modulus of immobile particles are also interpreted in details.

## Results

### Effective cooling rate

**Figure 1:**
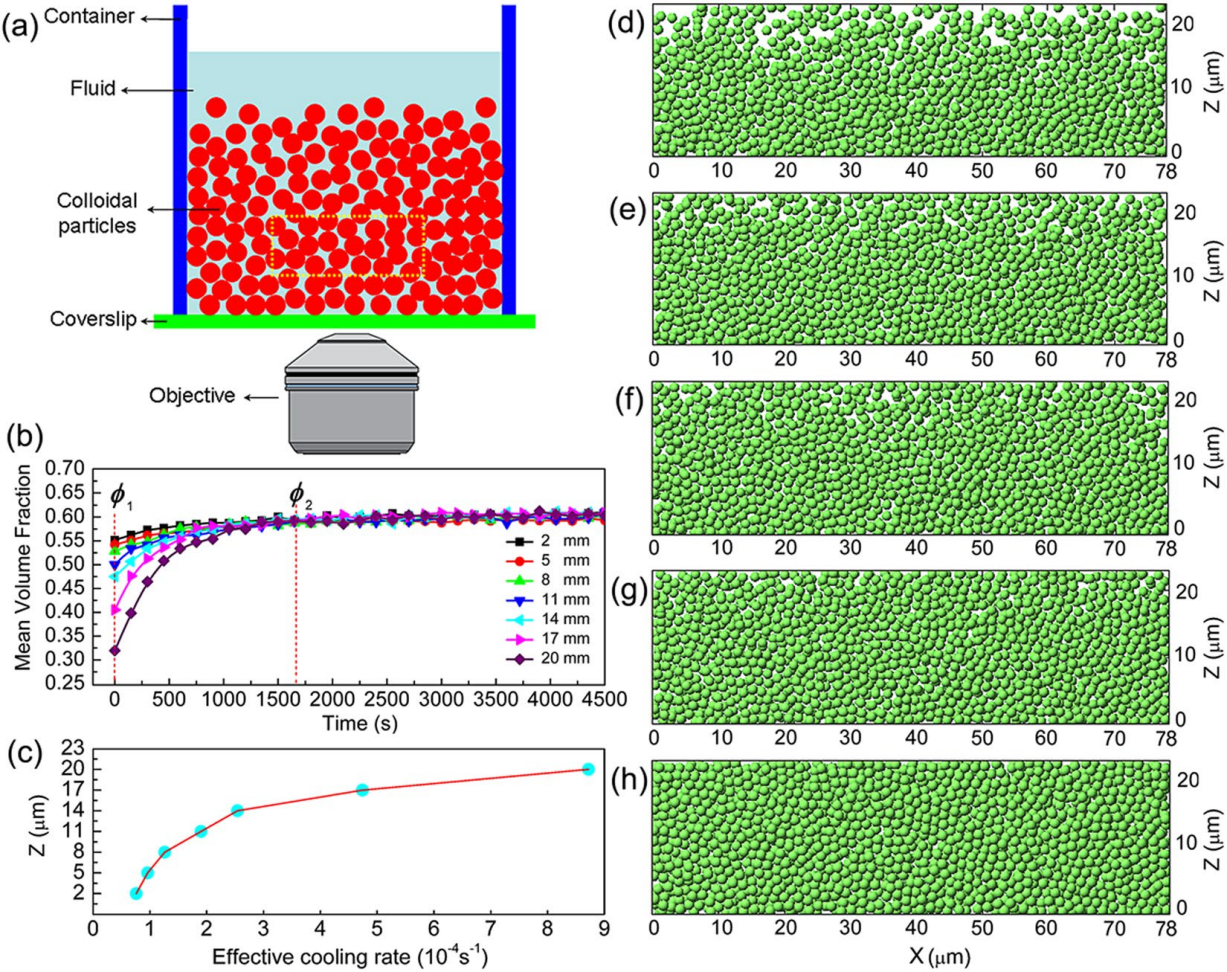
Effective cooling rate of colloidal glass. (**a**) The sketch of the experimental set-up. (**b**) The mean *ϕ* values of glass sections at different heights of colloidal glass. (**c**) ${\dot{T}}_{eff}$ of glass sections at different heights of colloidal glass. (**d**,**e**) Typical reconstructions of 3-μm-thick x-z sections of colloidal glass centered at y = 10 μm (**d**) at 0 s, (**e**) at 150 s, (**f**) at 300 s, (**g**) at 450 s, (**h**) at 1650 s.

In hard-sphere colloidal system, the viscosity, *η*, approaching the glass transition varies with volume fraction, *ϕ*, can be described as $\eta ={\eta }_{0}\exp [D\varphi /({\varphi }_{0}-\varphi )]$ ^17^. Correspondingly, the viscosity of a molecular liquid approaching the glass transition varies with temperature, *T*, can be described by Vogel-Fulcher-Tammann (VFT) equation, $\eta ={\eta }_{0}\exp [D{T}_{0}/(T-{T}_{0})]$ ^18^. Here, *D* is the fragility index, *η*_0_ is the viscosity at a high *T* or *ϕ*. *T*_0_ and *ϕ*_0_ results from a fit to where the viscosity would become infinite. The above two equations are similar in form, suggesting that *ϕ* plays the similar role in hard-sphere colloidal system as *T* in usual liquids^16,19^. In other words, the control parameter of the hard-sphere colloidal system is *ϕ* rather than *T*, and the effective temperature of hard sphere system is *T*_*eff*_ = 1*/ϕ* ^20^. Then, the effective cooling rate, ${\dot{T}}_{eff}$, of the hard-sphere system can be expressed as ${\dot{T}}_{eff}=(1/{\varphi }_{1}-1/{\varphi }_{2})/{\rm{\Delta }}t$, where *ϕ*_1_ and *ϕ*_2_ are the volume fractions of colloidal system at *t*imes *t*_1_ and *t*_2_, respectively; Δ*t* = *t*_2_ − *t*_1_ is the time interval be*t*ween *t*_1_ and *t*_2_. As described in experimental methods, we built a colloidal glass by sedimentation under gravity. The sketch of the experimental set-up is shown in Fig. 1(a). Figure 1(d–h), showing the typical process of the sedimentation of the colloidal system, are five reconstruct colloidal structures in 3-μm-thick x-z sections centered at y = 10 μm from the initial time to 1650 s. Along the z direction, the packing densities of the colloidal system vary a lot at the initial time (*t* = 0 s), but no apparent difference can be detected after 1650 s, suggesting that there may be ${\dot{T}}_{eff}$ difference along the height of the colloidal glass. To check this assumption, we calculated the ${\dot{T}}_{eff}$ at different heights. We measure the mean *ϕ* of the colloidal system in the 3-μm-thick x-y section centered at different heights. We determine the *ϕ* from the relationship $\varphi =n{V}_{0}$, where *n* is the number density of particles, and ${V}_{0}=4\pi {R}_{0}^{3}/3$ is the volume of colloidal particles with the radius *R*_0_. The number density *n* is calculated from the inverse of average Voronoi volume. The Voronoi volume of any individual particle is all of the space that is closer to the center of that particle than any other particle. Since the sum of all of the individual Vorono_*i*_ volumes *V*_*i*_ is equal to total system volume *V*_total_, the average Voronoi volume *V* can be given by $V=(\sum _{i=1}^{N}{V}_{i})/N={V}_{total}/N=1/n$, where *N* is the total number of particles and *n* is the number density of particles. Thus, the inverse of average Voronoi volume is a direct measure of the number density *n*. As shown in Fig. 1(b), all the mean *ϕ* values of glass sections at different heights increase with the increase of time and finally constant at about 0.60 after 1650 s, well into the glassy state^20^. We then chose the mean *ϕ* values at 0 s and 1650 s as *ϕ*_1_ and *ϕ*_2_, respectively. Then the calculated ${\dot{T}}_{eff}$ of glass sections at different heights are presented in Fig. 1(c). Clearly, the ${\dot{T}}_{eff}$ gradually increase with increasing the height, from 0.76 × 10^−4^ s^−1^ to 8.72 × 10^−4^ s^−1^. This gradual variation of ${\dot{T}}_{eff}$ along the height offers us a good chance to study the effect of cooling rate on the stress-bearing properties of colloidal glass.

### Shear modulus

**Figure 2:**
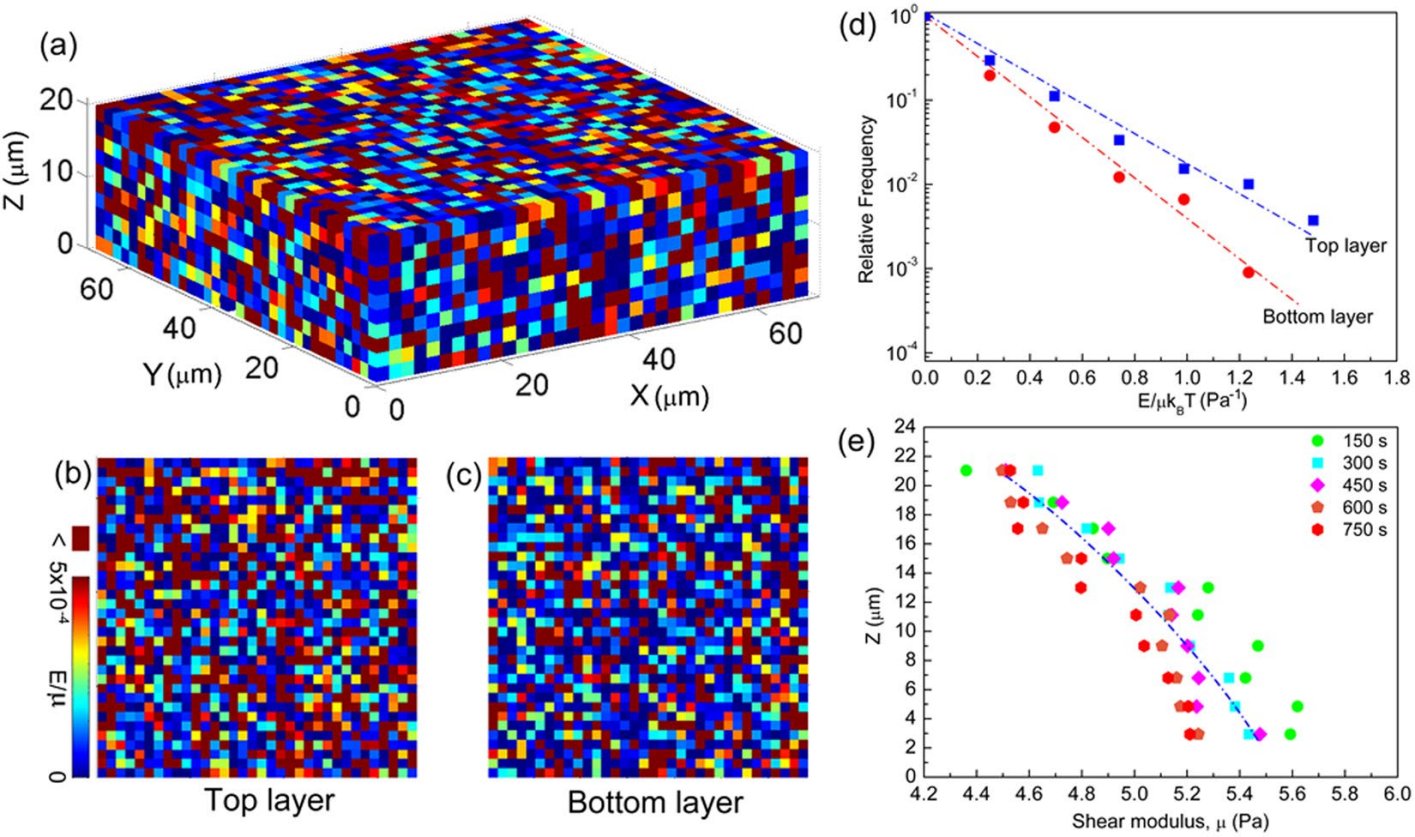
Shear modulus of colloidal glass. (**a**) A typical array of boxes showing the spatial distribution of *E*_xy_/*μ* magnitude that calculated from the *ε*_xy_ between 1650 s and 1800 s. (**b**) The top layer of the box array showing in (**a**–**c**) The bottom layer of the box array showing in (**a**). (**d**) The relative frequency as a function of *E*_xy_/*μk*_*B*_ *T* of each box shown in the (**b**) and (**c**). (**e**) The *μ* of 2-μm-thick x-y section centered at different heights of the colloidal glass for five time intervals. The reference time is 1650 s.

Shear modulus, *μ*, is a measure of the stiffness of a solid material. In the present study, we use *μ* as the representative parameter to monitor the softening of the colloidal glass. To measure the *μ* of the colloidal glass, we calculate the elastic energies associated with the distribution of shear strain and determine the relative frequency of the energies^21,22^. The strain field is determined by calculating the symmetric part of the best affine deformation tensor that describes the transformation of the nearest neighbor vectors^21,23^. Here, we focus on the shear component *ε*_xy_ of the strain tensor and illustrate its cumulative value with the reference state of the colloidal glass at 1650 s. We coarse-grained the *ε*_xy_ shear strain by dividing the glass sample into boxes with a side length *a* = 2 μm and calculate the total elastic energy in each box. The elastic energy *E*_xy_ associated with the *ε*_xy_ is *E*_xy_/*μ* = (1/2)(2*ε*_xy_^2^)*a* ^3^, where the elastic energy is normalized by *μ* ^21^. A typical array of boxes, showing the spatial distribution of *E*_xy_/*μ* magnitude that calculated from the *ε*_xy_ between 1650 s and 1800 s, is shown in Fig. 2(a). To observe the *E*_xy_/*μ* distribution more clearly, the top and bottom layers of the box array are shown in Fig. 2(b,c), respectively. Because of local thermal equilibrium, the elastic strain energies follow a Boltzmann distribution. The probability of elastic strain energies is exponentially distributed. Thus, the *E*_xy_ should occur with a probability of *P*(*E*_xy_) ∝ exp[−*μ*(*E*_xy_/*μk*_*B*_ *T*)]^21,24^. We then plot the relative frequency as a function of *E*_xy_/*μk*_*B*_ *T* of each box shown in the top and bottom layers in Fig. 2(d). The shear moduli of the top and bottom layers, obtained from the fit indicated by straight lines in Fig. 2(d), are *μ*_top_ = 4.4 Pa and *μ*_bottom_ = 5.6 Pa, respectively. To check the effect of cooling rate on the *μ*, we calculate the *μ* of 2-μm-thick x-y section centered at different heights of the colloidal glass for five time intervals. The reference time is 1650 s. As presented in Fig. 2(e), the overall trend of the *μ* is a gradual decrease with the rising of the height, indicating that the colloidal glass is softer under a higher cooling rate.

### Immobile particles

**Figure 3:**
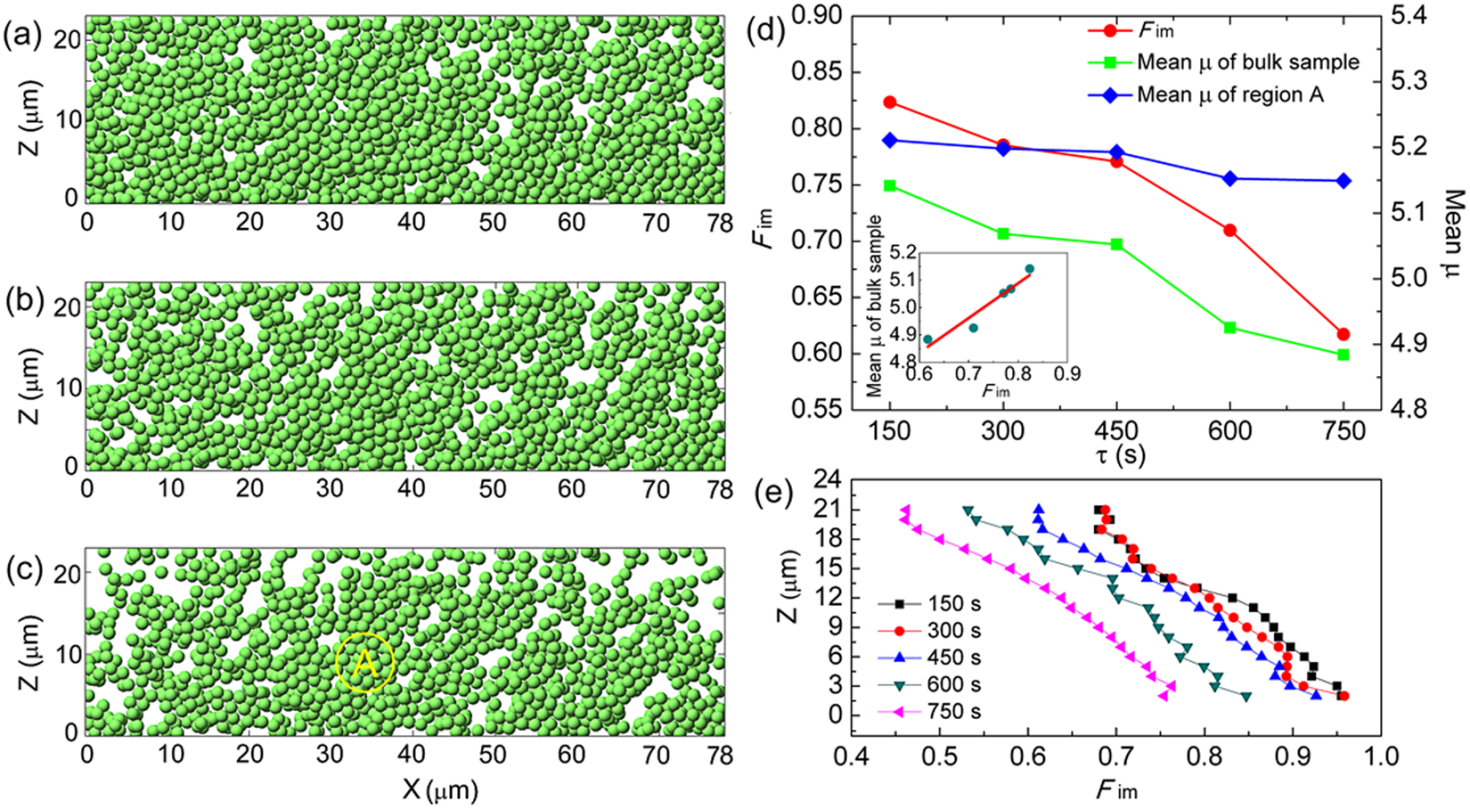
Immobile particles. (**a**–**c**) Typical reconstructs of immobile particles in 3-mm-thick x-z sections centered at y = 10 μm over (**a**) *τ = *150 s (1650–1800 s), (**b**) 450 s (1650–2100 s) and (**c**) 750 s (1650–2400 s). (**d**) *F*_im_ and mean *μ* of colloidal glass as a function of *τ*. (**e**) *F*_im_ of 2-μm-thick x-y sections centered at different heights of the colloidal glass for five time intervals. The reference time is 1650 s.

**Figure 4:**
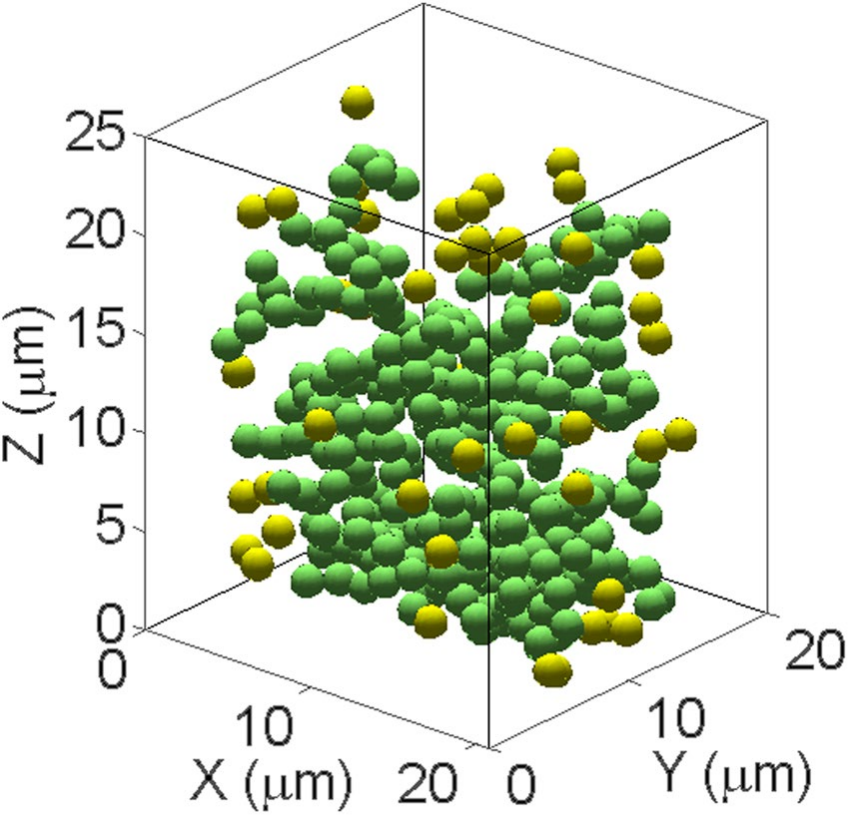
Typical reconstruct of immobile particles in a volume of 20 × 10 × 23 μm^3^ over 750 s (1650–2400 s). The green particles form a cluster percolating across the sample. Other isolate particles separating with the cluster are drawn yellow for clarity.

We determine the immobile particles by measuring Δ*N*(*τ*), which is the number of changes in each particle’s nearest neighbors over a time interval *τ*. The nearest neighbors to a given particle are considered to be all particles within the distance of the first minimum *r*_min_ of the radial distribution function (RDF). Then the immobile particle is identified as one for which Δ*N*(*τ*) = 0. Since the immobile and mobile particles determined by this method exist within any timescales, to observe the distribution of immobile particles clearly, we reconstruct three typical distribution of immobile particles over three arbitrary timescales *τ* = 150 s (1650–1800 s), 450 s (1650–2100 s) and 750 s (1650–2400 s) in 3-μm-thick x-z sections centered at y = 10 μm, as shown in Fig. 3(a–c). The immobile particles are shown strongly spatially correlated and exhibit extended clusters. To observe the distribution of immobile particles more clearly, we have reconstructed immobile particles in a typical volume of 20 × 10 × 23 μm^3^ over 750 s (1650–2400 s). As presented in Fig. 4, the green particles form a cluster percolating across the sample. Other isolate particles separating with this cluster are drawn yellow for clarity. As can be seen from Fig. 3(a–c), the number of immobile particles in the clusters decreases with the increasing time scale, as shown in Fig. 3(d) which plots the fraction of immobile particles *F*_im_ as a function of *τ*. Though the clusters of immobile particles break up on longer time scales, the clusters still percolate across the glass system, indicating that the system-spanning clusters of immobile particles may directly and quantitatively be connected with the stress-bearing properties of amorphous solids. To investigate the effect of cooling rate on the immobile particles, we calculate the *F*_im_ of 2-μm-thick x-y sections centered at different heights of the colloidal glass for five time intervals. The reference time is 1650 s. As presented in Fig. 3(e), the *F*_im_ gradually decreases with increasing the height, revealing that fewer immobile particles are formed in the colloidal glass under a higher cooling rate.

## Discussion

**Figure 5:**
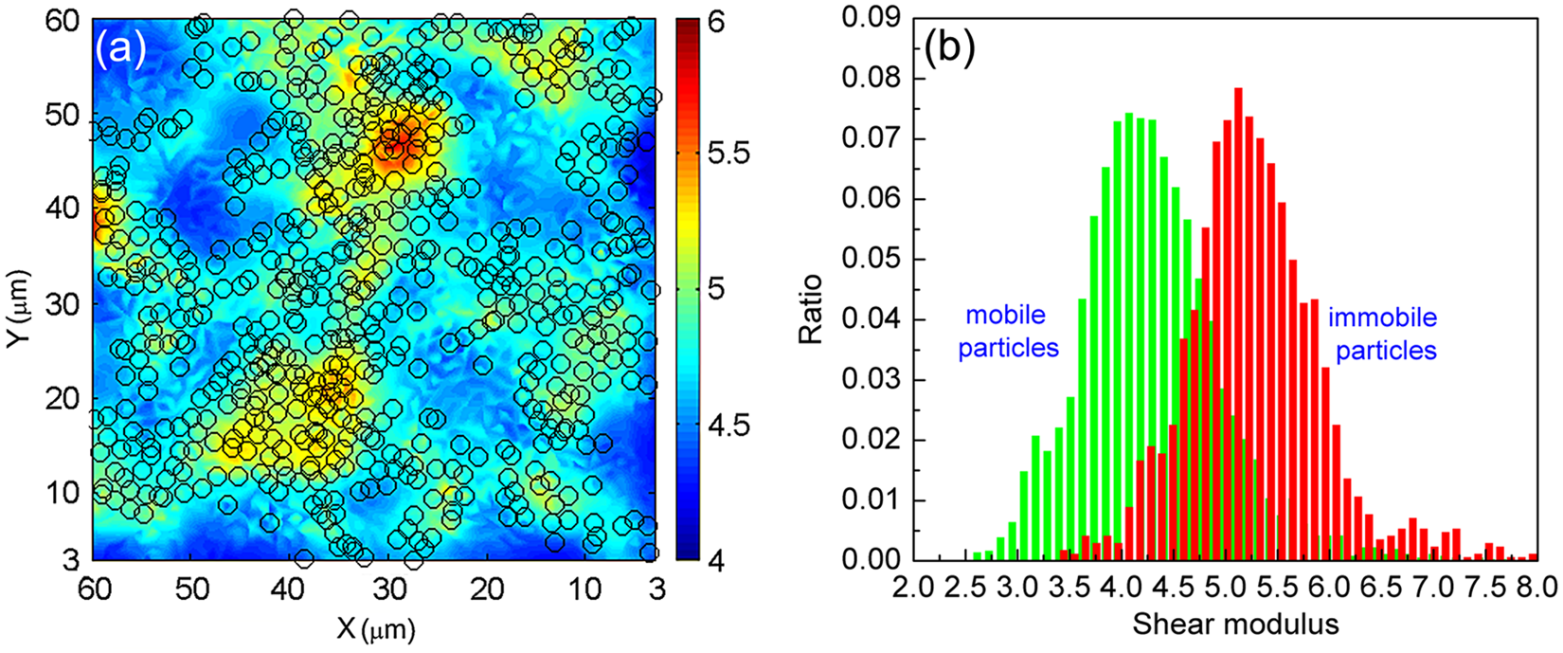
Contribution of immobile particles to shear modulus. (**a**) Colored contour plots of the *μ*_*i*_ of the top 3-μm-thick x-y glass section. The black circles plotted overlaid indicate the immobile particles determined at 2400 s with the reference state at 1650 s. (**b**) Histograms showing the shear modulus of the immobile and mobile particles in the entire glass sample. The immobile and mobile particles determined at 2400 s with the reference state at 1650 s.

So far, our results have shown solid evidences that the immobile particles may essentially correlate with the *μ*. As displayed in Figs 2(e) and 3(e), the *μ* and *F*_im_ show the same distribution trends along the height of colloidal glass. Another evidence is the dependence of the mean *μ* and *F*_im_ of the bulk glass sample on *τ*. As shown in Fig. 3(d) and its inset, an almost linear relationship between *μ* and *F*_im_ can be found. To provide a more direct evidence on the contribution of immobile particles to *μ*, we explore the local shear modulus *μ*_*i*_ of each colloidal particle in the top 3-μm-thick x-y section of colloidal glass. Since the strain fluctuation is induced by thermally activated relaxation, the fluctuation of shear strain component *ε*_xy,*i*_ of *i*-th colloidal particle is supposed to be excited on average with thermal energy *k*_*B*_ *T* in local thermal equilibrium^10,22^. Then the elastic strain energy *E*_xy_ of each colloidal particle is equal to the thermal energy *k*_*B*_ *T*, i.e., *k*_*B*_ *T* = (1/2)*μ*_*i*_(2 < *ξ*_xy,*i*_^2^ >)*V*_0_. Here, < *ξ*_xy,*i*_^2^ > is the time average of *ε*_xy,*i*_^2^ over 5 adjacent 150-s time intervals from 1650 s to 2400 s. Then the *μ*_*i*_ can be obtained as *μ*_*i*_ *= *(< *ξ*_xy,*i*_^2^ > ·*V*_0_)^−1^ *k*_*B*_ *T*. We show the colored contour plots of the _*μi*_ of the top 3-μm-thick x-y glass section in Fig. 5(a). The black c_*i*_rcles plotted overlaid on this figure indicate the immobile particles determined at 2400 s with the reference state at 1650 s. A clear correlation between the spatial distributions of immobile particles and larger *μ*_*i*_ is observed, supporting that the majority contribution to the shear modulus comes from the immobile particles. We further quantify the correlation between the shear modulus and immobile particles by plotting the histograms of shear modulus of the immobile and mobile particles in the entire glass sample. The immobile and mobile particles determined at 2400 s with the reference state at 1650 s. As can be seen from Fig. 5(b), the magnitudes of the shear modulus of the immobile particles (red bars) are significantly larger than those of the mobile particles, demonstrating that the immobile particles closely correlate with and provide the major contribution to the shear modulus. Therefore, the softening of the colloidal glass caused by higher cooling rate originates from fewer immobile particles formed under higher cooling rate.

Since both the immobile and mobile regions in a colloidal glass are structurally amorphous, one logical question would be why the immobile regions behave more stress bearing. According to previous studies, the glass transition from supercooled-liquid state to glassy state is intrinsically an increase in the relaxation time and the percolation of slow dynamic regions^25,26^. In this case, the immobile regions in the colloidal glass can be reasonably interpreted as those with much longer relaxation time and thus higher viscosity^27^. In other words, the immobile regions are“quasi-static”, and even flow and become softer if one could wait for a long time. This statement can be supported by variation of mean *μ* in an immobile particle region A indicated by the circle in Fig. 3(c). The dependence of mean *μ* of region A on *τ* is presented in Fig. 3(d), a slight but detectable decrease in the mean *μ* of region A can be found after waiting for 750 s. From this experimental evidence we can logically deduce that the immobile regions behave more elastically simply because the experimental time scale is much shorter than the relaxation times of immobile regions.

## Conclusions

We have built a colloidal glass with a cooling-rate gradient along its height as the model system to directly study the cooling-rate-induced softening of amorphous solids. Shear modulus is used as the representative parameter to monitor the stress-bearing properties of colloidal glass. The space-spanning immobile particles, whose population is sensitive to the cooling rate, are found to make the dominant contribution to the shear modulus. The softening of the colloidal glass caused by higher cooling rate originates from fewer immobile particles formed under higher cooling rates. Such immobile particles should also be present in other amorphous solids. The origins for the large shear modulus of immobile particles are attributed to the shorter experimental time scale comparing to the relaxation times of immobile regions.

## Methods

**Figure 6:**
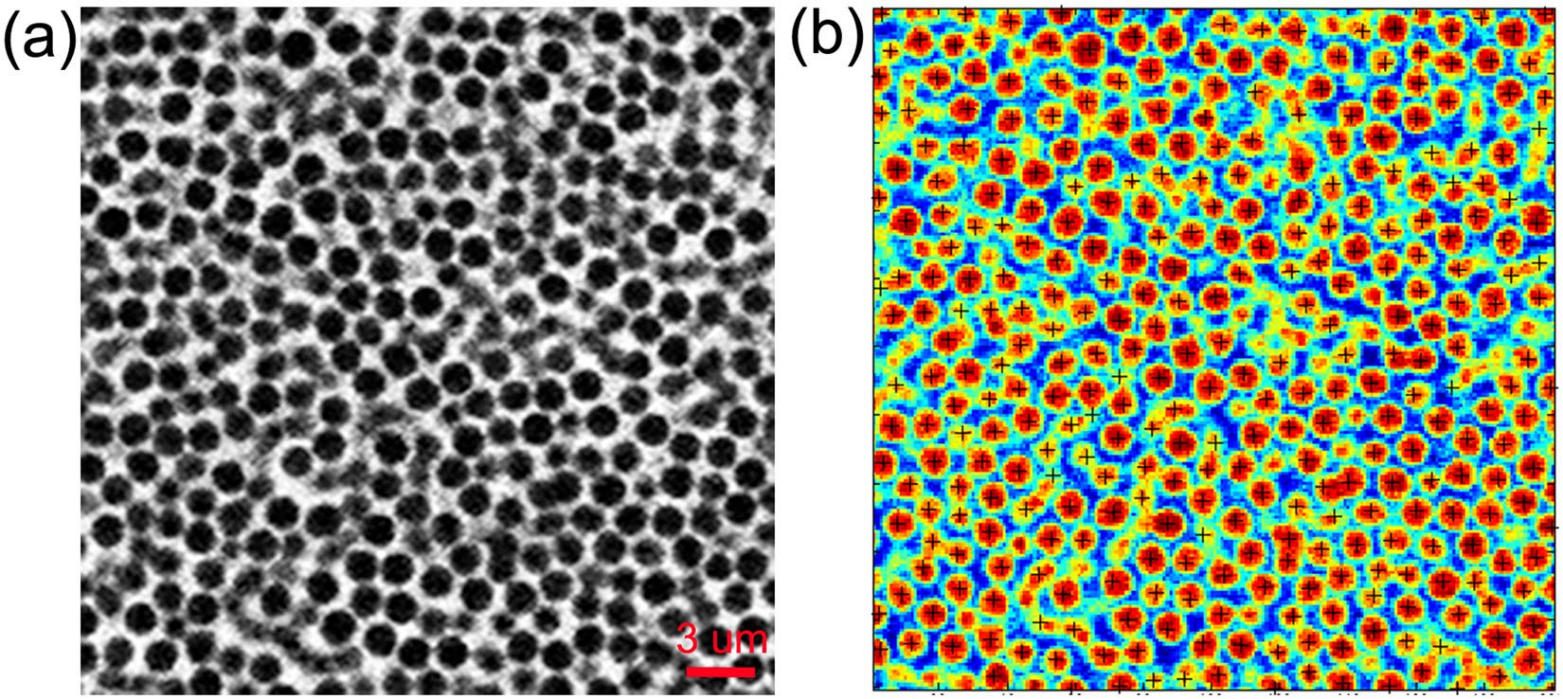
Particle locating. (**a**) A typical x-y slice of raw image acquired using the confocal microscope. (**b**) Particles found after the locating process, marked as black “+” superimposed on the same x-y slice.

The 1.55 μm diameter colloidal silica particles with a polydispersity smaller than 3.5% were used to prepare a colloidal system. More than 4 × 10^9^ colloidal particles were contained in this colloidal system. Following the experimental protocol used in reference^28^, the silica particles were suspended in a mixture of deionized water and dimethyl sulfoxide. The fluid phase has a viscosity of 1.6 mPas and matches the index of refraction of the silica particles. To make the particles appear as dark spots on a bright background under fluorescence microscopy, we dyed the solvent with fluorescein-NaOH solution^29^. The sample cell, composed of a metal tube with a diameter of 10 mm and glued onto a glass coverslip, is used to build colloidal system. The schematic of the sample cell is shown in Fig. 1(a). The density of colloidal particle is nearly twice larger than that of surrounding fluid, with the density difference of about Δ*ρ* ≈ 0.9 g/cm^3^. The colloidal structures were constructed by sedimentation under gravity due to the density difference. The Péclet number, *Pe*, for the sedimentation of colloidal system at room temperature *T* under gravity is about *Pe = *Δ*ρgR*_0_^4^/*k*_B_ *T* ≈ 0.8, where *g* is the acceleration due to gravity. The sedimentation of the colloidal system starts from the initial mean volume fraction of about *ϕ*_0_ ≈ 4%. The dimensionless deposition flux is about *ϕ*_0_ *Pe* ≈ 0.032, corresponding to a very high quenching rate^29^. Thus, crystals are not observed in our final colloidal glass with the volume fraction of roughly 0.60 that formed under gravity^30^. The sample cell mounts on a high-speed confocal microscope, which allows for imaging the 3D suspension structure. The confocal microscope we used in this study is the Leica SP5 laser point-scanning confocal microscope. This microscope has a “z-galvo” mode, in which the objective keep stationary and the sample stage moves. The z-galvo stage can provide faster and accurate scan steps along the z direction. We acquired three-dimensional scans of our sample yielding a 77 × 77 × 23 μm^3^ observation volume for each image stack. Each image stack takes 150 s. The total height of the colloidal glass is about 160 μm. In order to obtain clear images from the confocal microscope, we select an observation region at the bottom part of the colloidal glass, as indicated by the rectangle in Fig. 1(a). We determine individual particle positions in three dimensions with an accuracy of about 0.03 μm in the *x* and *y* directions and 0.05 μm in the *z* direction. In order to avoid possible boundary effects, all image stacks were taken far from walls of the sample cell. The distance from the bottom of the observation volume to the coverslip (the closest wall) is 30 μm. We located the particles from the raw confocal images in 3D by using standard particle locating software^31^, which was based on the algorithm used to locate particles in 2D systems^32^. The particle locating starts from loading the raw images into Matlab. Then the raw 3D images are bandpass-filtered to remove high-frequency noise and subtract any overall intensity gradients in the background. Then the compact “bright spots” corresponding to particles can be located through the feature finding software from the filtered image. The example of this locating process is described in Fig. 6 in this response letter, in which Fig. 6(a) is a typical x-y slice of raw image. Figure 6(b) shows the particle positions found after the locating process, marked as black “+” superimposed on the same x-y slice shown in Fig. 6(b). We convert the 3D image stacks into a series of coordinates of particle locations through particle locating. Based on the 3D particle coordinates that got from particle-locating procedure, typical reconstructions of 3-μm-thick x-z sections of the colloidal glass is presented in Fig. 1(d–h).
